# How to approach aortic valve disease in the elderly: a 25-year retrospective study

**DOI:** 10.5830/CVJA-2014-051

**Published:** 2014

**Authors:** Ebuzer Aydin, Ozge Altas Yerlikhan, Behzat Tuzun, Yucel Ozen, Sabit Sarikaya, Mehmet Kaan Kirali

**Affiliations:** Kartal Kosuyolu Training and Research Hospital, Istanbul, Turkey; Kartal Kosuyolu Training and Research Hospital, Istanbul, Turkey; Kartal Kosuyolu Training and Research Hospital, Istanbul, Turkey; Kartal Kosuyolu Training and Research Hospital, Istanbul, Turkey; Kartal Kosuyolu Training and Research Hospital, Istanbul, Turkey; Kartal Kosuyolu Training and Research Hospital, Istanbul, Turkey

**Keywords:** aortic valve replacement, elderly, surgery, mortality

## Abstract

**Objective:**

In the last decade, the number of elderly patients suffering from aortic valve disease has significantly increased. This study aimed to identify possible factors that could affect surgical and long-term outcomes in the light of a literature review regarding the management of aortic valve disease in the elderly.

**Methods:**

Between January 1990 and December 2012, a total of 114 patients (64 males, 50 females; mean age 76.6 ± 3.6 years; range 70–87 years) with aortic valve replacement (AVR) alone, or combined with coronary artery bypass grafting (CABG) or mitral surgery in our hospital, were retrospectively analysed.

**Results:**

In-hospital mortality was seen in 19 patients. The major causes of in-hospital mortality were low-cardiac output syndrome in eight patients (42.1%), respiratory insufficiency or infection in six (31.5%), multi-organ failure in four (21%), and stroke in one patient (5.2%). The main postoperative complications included arrhythmia in 26 patients (22.8%), renal failure in 11 (9.6%), respiratory infection in nine (7.9%), and stroke in three patients (2.6%). The mean length of intensive care unit and hospital stays were 6.4 ± 4.3 and 18 ± 12.8 days, respectively. During follow up, late mortality was seen in 28 patients (29.4%). Possible risk factors for long-term mortality were type of prosthesis, EuroSCORE ≥ 15, postoperative pacemaker implantation, respiratory infection, and haemodialysis. Among 65 long-term survivors, their activity level was good in 53 (81.5%) and poor in two.

**Conclusions:**

Our study results demonstrated that an individually tailored approach including scheduled surgery increases short- and long-term outcomes of AVR in patients aged ≥ 70 years. In addition, shorter cardiopulmonary bypass time may be more beneficial in this high-risk patient population.

## Abstract

The life expectancy of European and American populations has been steadily increasing, now exceeding 80 years of age. Over the past decade in Turkey, a modest increase has been achieved with people now reaching 76 years.^1^ In response to increased lifespan, aortic valve replacement (AVR) has become widely accepted in elderly patients.

Isolated AVR has been associated with an acceptable low surgical mortality rate, with improved long-term survival and quality of life.^2^ Despite all improvements, concomitant procedures and associated co-morbidities may result in high-risk surgery, which led us to consider a transcatheter approach in these patients.

In the last decade, the number of elderly patients aged 80 years or older suffering from aortic valve disease has significantly increased. In this study, we aimed to identify possible factors that may affect surgical and long-term outcomes in the light of a literature review regarding the management of aortic valve disease in the elderly.

## Methods

This retrospective study included a total of 114 patients (64 males, 50 females; mean age 76.6 ± 3.6 years; range 70–87 years) with AVR alone, or combined with coronary artery bypass grafting (CABG) or mitral valve surgery, admitted between January 1990 and December 2012. The study was conducted in accordance with the principles of Declaration of Helsinki. The study protocol was approved by the institutional review board (IRB) of Kartal Kosuyolu Training and Research Hospital (IRB no: 538.38792-514.10-9472). Informed consent, which was obtained from the patients, was confirmed by the IRB.

Bileaflet prostheses were mostly used, based on our experience with mechanical valve implantation and due to the poor socio-economic status of the country in those years. During 2012, all accessible survivors were questioned to obtain data regarding their health status, the presence of chest pain, functional grades of dyspnoea [New York Heart Association (NYHA) class], and quality of life. In total, 98.9% of the survivors (*n* = 64) completed follow up through out-patient clinic visits or phone interviews.

Adverse events were defined according to the guidelines for reporting morbidity and mortality after cardiac valvular operations.^3^ Surgical mortality was defined as any death, irrespective of cause, occurring within 30 days of surgery in or out of hospital,^4^ and long-term mortality was defined as any death occurring 30 days or more after surgery.^5^ Postoperative disease progression was defined as bleeding, poor cardiac status, renal failure (transient or permanent need of haemodialysis), neurological events, and prolonged duration of ventilatory support/intensive care unit (ICU).

Data on the pre-, intra- and postoperative periods were obtained from hospital charts. Of 95 hospital survivors, 47 visited the out-patient clinic on a regular basis.

## Pre-operative patient characteristics

The study consisted of 114 patients, including 22 (19.3%) aged ≥ 80 years. The mean age was 76.6 ± 3.6 years (range 70–87). Baseline demographic characteristics and clinical data are presented in Table 1.

**Table 1:** Baseline demographic characteristics and clinical data

	*Number*	*%*
Median age (years)	76.6 ± 3.6	(70–87)
Women/men	50/64	43.8/56.1
Hypertension	88	77.2
Diabetes	26	22.8
Chronic pulmonary disease	26	22.8
Pulmonary hypertension	8	7
Cerebrovascular disease	7	6.1
Peripheral vascular disease	1	0.9
Coronary artery disease	28	24.5
Chronic renal failure	11	9.6
Dyspnoea	75	65.8
NYHA		
Class II	14	12.3
Class III	81	71.1
Class IV	17	14.9
Angina pectoris	56	49.1
Previous MI	33	28.9
EuroSCORE < 15	91	79.8
> 15	23	20.2

NYHA: New York Heart Association; MI: myocardial infarction.

A total of 110 patients (87.7%) had at least one or more extra-cardiac co-morbidity, such as pulmonary disease (*n* = 26), cerebrovascular accident (*n* = 7), peripheral artery disease (*n* = 1), or renal failure (*n* = 11). Coronary angiography revealed significant lesions in 28 patients (24.5%), including five with left main coronary artery disease (LMCA). Twenty-three patients (20.2%) had logistic EuroSCORE ≥ 15. Ninety-eight patients (86%) had NYHA (LMCA) class III–IV symptoms, whereas 16 patients (14%) had clinical manifestations of congestive heart failure.

Seventeen patients (14.9%) had chronic atrial fibrillation, including two with a pacemaker. The common pathology of the valve was aortic stenosis in 97 patients (85.1%), while 22 (19.3%) had concomitant aortic regurgitation. Only 17 patients had uncomplicated pure regurgitation. Transthoracic echocardiography showed that 10.5% of patients (*n* = 12) had poor left ventricular function, defined as LVEF < 40% (Table 2).

**Table 2:** Pre-operative measurements

	*Number*	*%*
Pre-operative AF	17	14.9
Pacemaker	2	1.8
Echocardiography		
Aortic stenosis (AS)	97	85.1
Aortic regurgitation (AR)	22	19.3
LVEF (mean)	56.9 ± 9.3	(35–78)
LVEF < 40%	12	10.5
Aortic area (cm^2^)	0.8 ± 0.3	(0.4–1.35)
Max gradient (mmHg)	81.2 ± 25	(0–160)
Mean gradient (mmHg)	50.3 ± 16.4	(0–110)

AF: atrial fibrillation; LVEF: left ventricular ejection fraction.

## Surgical data

A median sternotomy was performed in all patients. Cardiopulmonary bypass (CPB) equipment was uniform: systemic moderate hypothermia was employed along with antegrade ± retrograde isothermic blood perfusion in case of aortic valve regurgitation. Three patients (2.6%) had a history of previous CABG surgery.

A mechanical valve was implanted in all but 22 patients (19.2%) received bioprosthetic valves. The proportion of bioprothetic valve replacement was higher in patients aged ≥ 80 years (27.2 vs 17.3%). We mostly used bileaflet prostheses based on our experience with mechanical valve implantation and due to the poor socio-economic status of the country in those years.

Isolated aortic valve replacement was performed in 61 patients (53.5%), whereas 29 underwent concomitant CABG surgery, 12 received mitral valve surgery, and 13 patients received interpositional graft replacement of the ascending aorta, including a flanged Bentall de Bono procedure due to type I dissection in one patient. The mean CPB time was 139.9 ± 73.7 min, while the mean aortic cross-clamp time was 96 ± 41.5 min (Table 3).

**Table 3:** Intra-operative data

	*Number*	*%*
Re-operation	6	5.2
Isolated AVR	61	53.5
AVR+ concomitant surgery		
CABG	29	25.4
Mitral valve surgery	12	10.5
CABG + MVR	1	0.8
Tubular graft interposition	13	11.4
Valve size (mm)	2.89	
Aortic cross clamping time (min)	96 ± 41.5	(26–240)
Cardiopulmonary bypass time (min)	139.9 ± 73.7	(46–480)
Postoperative inotropic support	60	52.6
IABP support	12	10.5

AVR: aortic valve replacement; CABG: coronary artery bypass grafting; MVR: mitral valve replacement; IABP: intra-aortic balloon pump.

Sixty patients (52.6%) required inotropic support for haemodynamic recovery, either in the operating room or during the postoperative period. Twelve received intra-aortic balloon pump (IABP) support. The chi-square test showed a significant correlation between the logistic EuroSCORE and inotropic support. In addition, 19 patients (82.6%) with EuroSCORE ≥ 15 needed pharmacological support (*p* = 0.001). Patients with LMCA stenosis (*p* = 0.008), NYHA ≥ 3 (*p* = 0.033) and EuroSCORE ≥ 15 (*p* = 0.002) were found to be correlated for the use of IABP.

International normalised ratio (INR) levels were measured daily and postoperative anticoagulant therapy was administered with oral sodium warfarin in all patients. Three-month therapy following bioprosthetic valve replacement was prescribed.

## Statistical analysis

Statistical analysis was performed using the SPSS software v12.0 (SPSS Inc, Chicago, IL, USA). Continuous variables are expressed as mean ± standard deviation or percentages. The Student’s *t*-test and Mann–Whitney *U*-test were used to compare differences among the variables.

One-way analysis of covariance (ANOVA) and the Tukey *post hoc* analysis were used to compare three or more normally distributed samples. In the case of abnormally distributed data, the Kruskal–Wallis test was used for more than three samples, whereas the Mann–Whitney *U*-test was used to compare two samples based on the adjusted Bonferroni correction. The cross tabulation table was used to compare categorical variables (chi-square, Fisher, Mantel–Haenszel test).

All variables were initially tested individually by univariate analysis. Then, variables with a *p*-value of ≤ 0.25 in univarate analysis were applied into the logistic regression model to identify independent predictors for mortality. Late survival rates were calculated using the Kaplan–Meier method and statistical significance was calculated with the log-rank test. A *p*-value of < 0.05 was considered statistically significant.

## Results

The mean follow up was 48.7 ± 50.8 months (range 0–240). Early postoperative complications are listed in Table 4. Arrhythmias occurred in 26 patients (22.8%), of whom 13 had atrial fibrillation. All received medical therapy. The other 13 (11.4%) needed permanent pacemaker implantation. Despite optimal selection of the prosthesis to minimise the incidence of pacemaker implantation, we found no correlation among peri-operative risk factors, including prosthesis type (*p* = 0.457). However, pre-operative values of NYHA (*p* < 0.001) and logistic EuroSCORE (*p* = 0.023) were found to be significantly correlated with peri-operative risk factors.

**Table 4:** Postoperative complications

*Variable*	*Number*	*%*
Arrhythmia	26	22.8
Pacemaker implantation	13	11.4
Prolonged mechanical ventilation	30	26.3
Bleeding total (ml)	641 ± 480.56	
Re-operation		
Bleeding	3	2.6
Tamponade	3	2.6
Cerebrovascular accident	3	2.6
Renal failure	11	7.9
Haemodialysis (permanent)	4	3.5
Pulmonary failure	9	7.9

Renal failure was present in 11 patients (9.6%), of whom seven patients needed transient haemodialysis after surgery. Four (3.5%) required long-term haemodialysis. Among 30 patients (26.3%) requiring prolonged mechanical ventilation (> 24 hours), nine (7.9%) had respiratory infection. There was a significant correlation between LMCA stenosis and infection (*p* = 0.049).

Three patients (2.6%) developed cerebrovascular accident and two recovered fully before hospital discharge. Six patients needed re-operation, of whom three were operated on due to excessive bleeding. The rest were operated on for cardiac tamponade. The mean length of ICU and hospital stay was 6.4 ± 4.3 and 18 ± 12.8 days, respectively.

In-hospital mortality was seen in 19 patients (16.7%). The mortality rate was 8.7% in 10 patients who underwent isolated AVR. The mean time to death after surgery was 17 ± 15.61 days (range 0–48 days). The major causes of in-hospital mortality were low-cardiac output syndrome in eight patients (42.1%), respiratory insufficiency or infection in six (31.5%), multi-organ failure in four (21%), and stroke in one patient (5.2%). Univariate analysis revealed the following variables to be associated with operative mortality: LMCA stenosis (*p* = 0.032), NYHA ≥ III (*p* = 0.002) and EuroSCORE ≥ 15 (*p* < 0.001).

During the follow-up period, 28 late deaths (29.4%) occurred. A total of 98.9% completed the follow-up period. Based on the univariate analysis, possible risk factors for long-term mortality were type of prosthesis (*p* = 0.037), EuroSCORE ≥ 15 (*p* = 0.013), postoperative pacemaker implantation (*p* = 0.008), respiratory infection (*p* = 0.004), and haemodialysis (*p* = 0.004). Mortality rate was higher in the mechanical valve group. Multivariate analysis identified the following variables as independent predictors of mortality: cross-clamp time (*p* = 0.043) and CPB time (*p* = 0.033).

Furthermore, although cerebrovascular accidents, either from intracranial haemorrhage or ischaemic stroke, were the leading cause of death (*n* = 8). Respiratory failure (*n* = 7) and cardiovascular disease (*n* = 6) accounted for 46.4% of the late mortalities. Three patients died from renal insufficiency, while one had a neoplasm, one had mesenteric ischaemia, and two patients suffered from sudden death.

**Fig. 1.**
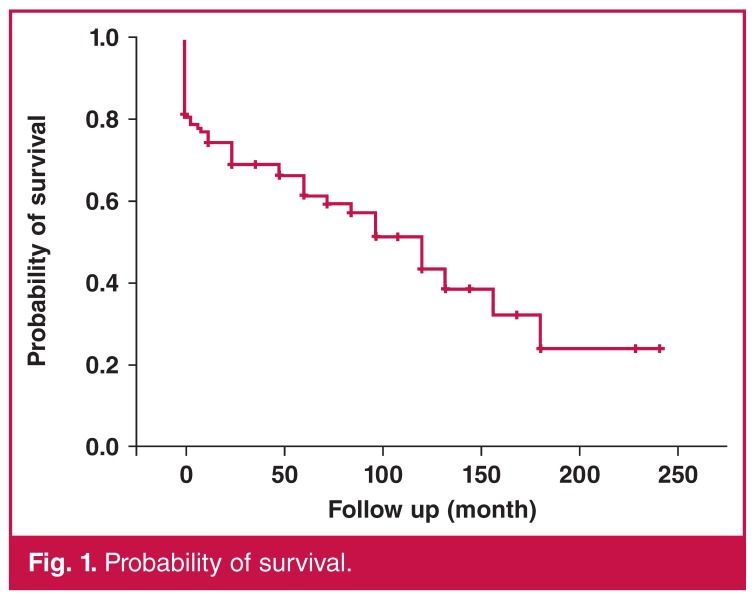
Probability of survival.

**Fig. 2.**
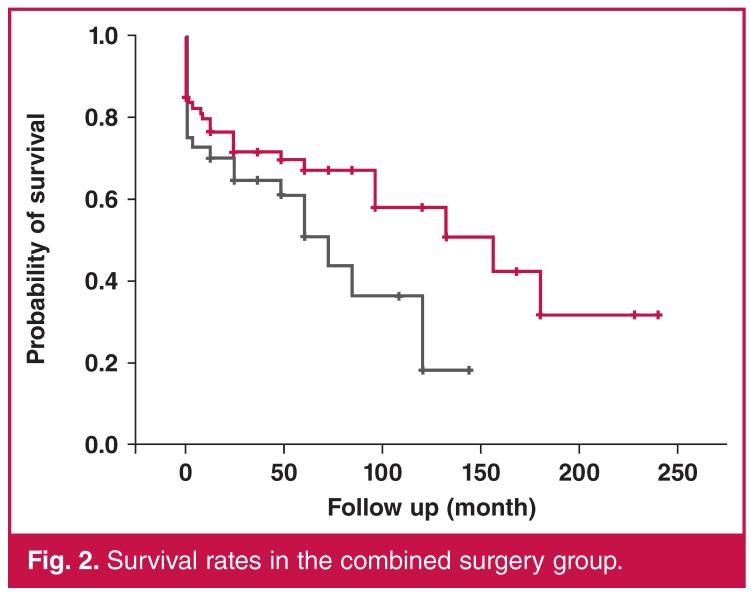
Survival rates in the combined surgery group.

One, three, five, 10 and 15-year survival rates were 76.2 ± 4.12, 69.03 ± 4.44, 61.40 ± 5.13, 43.48 ± 7.42 and 24.15 ± 9.65%, respectively (Fig. 1). Patients with combined surgery showed lower survival rates (log-rank, *p* = 0.0498) (Fig. 2).

During follow up, late complications were intracranial haemorrhage in one patient, stroke in one patient and re-operation for vegetations and paravalvular leakage in two patients (at 10 years and one year after the initial surgery, respectively).

The patients were questioned on symptom relief and an active lifestyle. Among 65 long-term survivors, activity level was good in 53 (81.5%) and poor in two (3.1%). The patients reported improved quality of life compared to their pre-operative status. We were unable to reach 10 patients to determine their activity levels.

## Discussion

Since 1970, men and women worldwide have gained slightly more than 10 years of life expectancy overall, but they spend more years living with injury and illness. Non-communicable diseases, such as cancer and heart disease, have become the dominant causes of death and disability worldwide.^6^

With the introduction of improved surgical techniques and prolonged life expectancy, an increasing number of elderly people are considered candidates for valve surgery. According to the Euro Heart Survey, intervention was rejected in up to 33% of patients, despite their severe symptomatic aortic stenosis (AS) status.^7^ However, the natural prognosis of severe AS is associated with a life expectancy of less than five years.^8^,^9^ In our study, we found that mortality rates in elderly patients (≥ 70 years) who underwent timely aortic valve operations were very low. This encourages us to refer especially the elderly with aortic stenosis for surgery.

Several studies have showed that valve replacement can be performed with an acceptable mortality rate and high long-term survival rate.^10^-^12^ Kohl *et al.*^13^ reported their operative mortality rate to be 13%, which was increased to 24% with combined surgery.^13^ In our study, in-hospital mortality was 8.7% in patients with isolated AVR and 16.7% in patients with combined surgery. In addition, early reports in the elderly have shown mortality rates of 2–10% for isolated AVR.^14^,^15^

Concomitant CABG was also identified as an independent predictor in a clinical series.^16^ However, in a study including 450 patients aged ≥ 80 years, Unic *et al.*^17^ showed that concomitant CABG did not affect the late survival rate.

In our study, we did not find any correlation between combined surgery and mortality rate. Even though LMCA stenosis, NYHA ≥ III and EuroSCORE ≥ 15 were associated with early mortality in the univariate analysis, multivariate logistic regression analysis revealed that the only risk factor associated with surgical mortality was CBP time, as anticipated. In other words, simple operations with shorter CPB times may be more beneficial than better complex operations with longer CPB times in high-risk patients.

In addition, our study findings confirmed that patients undergoing combined surgery with concomitant CABG showed lower long-term survival rates compared to surgical mortality rates, from 16.7% post operatively to 13.3% long-term survival. This is noticeably lower than the 24% early mortality that was reported by Kolh *et al.*^18^ Kurlansky *et al.*^16^ also identified concomitant CABG as a predictor of mortality; however, they showed the improvement in quality of life in the long term.

In our study, mitral valve surgery was associated with an increased mortality rate (30.1%). This was consistent with a number of previous studies.^15^,^19^ It has been reported that AVR can be performed in elderly patients with an acceptable mortality rate, high long-term survival rate and functional improvement.^14^,^20^ One-, three- and five-year survival rates were 76.2 ± 4.12, 69.03 ± 4.44 and 61.40 ± 5.13%, respectively. However, these rates need to be confirmed.^13^,^14^

The low survival rates in our study can be attributed to multiple factors, including that 87.7% of patients had extracardiac co-morbidities; 10.5% had poor ejection fraction, and 86% were in NYHA class ≥ III. Postoperative pacemaker, respiratory infection and haemodialysis were predictors for late mortality, while aortic cross-clamp time and CPB time were found with multivariate analysis to be independent predictors of mortality. These results suggest that combined surgery entails prolonged ischaemic time, leading us to tailor an appropriate surgical strategy for each patient.

The use of bioprosthetic valves, which allow for implantation of larger prostheses, was lower than in previous studies. The early experiences of Peterseim *et al.*^21^ reported that bioprostheses should be considered in patients with a number of co-morbidities or aged ≥ 65 years. In a retrospective study, however, Silberman *et al.*^22^ reported that the selection of valve replacement device should be based on life expectancy, patient preference, lifestyle and surgery-related complications.

The limitations of the present study include missing information due to limited data collection, as it was a retrospective study, and missed regular out-patient visits. Although 98.9% of patients completed the follow-up period, less attention was paid to the quality of life. Among long-term survivers, only two patients had poor activity levels. However, the Short form 36 health survey should be completed for further investigation of long-term quality of life.

## Conclusion

It is obvious that we need more surgical experience on elderly patients. Our study results demonstrate that an individually tailored approach including scheduled surgery increased short-and long-term outcomes of AVR in patients aged ≥ 70 years. In addition, shorter cardiopulmonary bypass time may be more beneficial in this high-risk patient population.

Although several issues should be considered for elderly patients undergoing cardiac surgery, including socio-economic factors, the possible benefits of surgery should not be ignored in patients with aortic valve disease who are eligible for surgery. In addition, in the presence of combined cardiac procedures, a hybrid approach or transcatheter aortic valve implantation with isolated conventional AVR may be an alternative in high-risk patients.
